# Supra-Pubic Cystotomy

**Published:** 1883-07

**Authors:** Joseph W. Stickler

**Affiliations:** Visiting Physician and Curator at the Orange Memorial Hospital, and Lecturer on Comparative Surgery and Skin Diseases at the Columbia Veterinary College


### ART. XXII.—SUPRA-PUBIC CYSTOTOMY.

BY J. W. STICKLER, M. S., M. D.,

*Visiting Physician and Curator at the Orange Memorial Hospital, and Lecturer on Comparative Surgery and Skin Diseases at the Columbia Veterinary College.*

THERE are diseases and traumatic lesions which may so affect the urethra, that the passage of urine is made impossible either temporarily or permanently. When the former condition obtains, the question arises, how can the bladder be evacuated for a sufficiently long time to make it possible to restore the function of the urethra by local surgical interference? It occurred to me that an artificial outlet for the urine might be established, by making an opening into the bladder through the abdominal walls. To secure this result I performed the following operation upon three rabbits. Having etherized them, they were in turn placed upon their backs and after cutting off enough hair from the abdomen to expose the skin immediately in advance of the pubic bones, I made an incision about one inch and a half long in the median line of the abdomen, cutting through the various muscles and fasciæ so as to bring the base of the bladder into view. On separating the lips of the wound the intestines at once filled the opening, but on gently pushing them aside, the bladder was comparatively easily secured, and after being well drawn forward with a pair of thumb forceps, was incised to the extent of about one-third of an inch, the line of incision extending from the point at which the forceps were applied, toward the neck of the viscus. To maintain the cut surface of the bladder in opposition with abdominal aperture, several silk sutures were used, care being taken to leave no openings between the sutures to admit of leakage into the peritoneal cavity. To assure myself that the opening into the bladder was complete and unobstructed in each case I passed a large sized probe into it. There were no dressings used, the animals being being allowed to run freely in a large box prepared for them. After the operations there was a constant flow of urine, but union was not materially retarded, for on the fifth day

I removed the sutures and found the bladder firmly adherent to the abdominal wound, and the artificial opening unobstructed. In one case there was an excessive growth of granulations about the lips of the wound, but these were destroyed, and a healthy process substituted by the application of nitrate of silver. About one month from date of operation the case just referred to began to suffer from prolapse of the bladder, due to a giving away of the stitches in the abdominal wound adjacent to the opening, the natural support of the organ being in this way very greatly weakened. The irritation to which the bladder was subjected by coming into contact with the floor of the box, together with the constant flow of acid urine over its already inflamed mucous membrane finally caused the death of the animal. In the other two cases the openings remained perfectly patent about one month and then became smaller and smaller, entirely closing at the expiration of the second month. There was not the slightest evidence of peritonitis in any of these cases for there was neither marked elevation of temperature, tenderness on palpation, or tympanitic distention of the abdomen. This may be accounted for by the fact the lower animals are not so liable to peritoneal inflammation, after surgical injury or accidental trumatism, as man, but the fact stands, that the base of the bladder can be opened and an artificial urinary outlet secured with very little risk to the patient. In man the anterior surface of the bladder is without peritoneal covering, and in the horse that part of the viscus which rests upon the floor of the pelvis has no serous coat, hence when operated upon, would be less liable to be followed by peritonitis. Infiltration of urine into the cellular tissue of the abdomen, or into the peritoneal cavity, is rendered unlikely if proper care be taken in adjusting the sutures. In case the opening remains patent longer than is requisite, it may be closed by an operation such as adopted for the cure of a urinary fistula of spontaneous origin. Supra-pubic Cystotomy might also be performed in order to remove from the bladder a foreign body which could not be withdrawn through the urethra. In such an event the wound would be closed immediately after the operation, and union effected without delay, the urine passing at once through the natural passage. Such an operation as the one just described would not be performed when it could be substituted by a simpler one, but there are conditions which would speedily jeopardize life, were it not for the fact that the bladder can be opened without special danger to life, at a point where there is ample room for the removal of a foreign body, or the formation of a temporary outlet for the urine. So far as the functional disturbance is concerned, it seems to be so inconsiderable as to be practically unworthy of consideration at least from a therapeutic standpoint.

